# One case report of low-segment giant uterine fibroids removed combined with cesarean section for delivery

**DOI:** 10.1097/MD.0000000000043874

**Published:** 2025-08-29

**Authors:** Jianfen Wang, Chengzhao Shu, Yan Dong

**Affiliations:** a Gansu University of Chinese Medicine, Lanzhou, Gansu Province, China; b Gansu Maternal and Child Health Care Hospital, Lanzhou, Gansu Province, China.

**Keywords:** cesarean section, giant uterine fibroid, pregnancy, pregnancy outcome

## Abstract

**Rationale:**

Large uterine fibroids in specific locations (e.g., lower uterus) pose significant controversy regarding removal during cesarean section (C-section) due to surgical difficulty, bleeding risk, and maternal/fetal safety concerns. This case addresses the challenge of a huge fibroid completely blocking the birth canal, preventing standard C-section.

**Patient concerns:**

A 35-year-old woman at 35 + 1 weeks presented with poorly controlled hypertension for 9 weeks, diagnosed as chronic hypertension with superimposed preeclampsia and a large uterine fibroid.

**Diagnoses:**

Chronic hypertension accompanied by preeclampsia, pregnancy with uterine fibroids.

**Interventions:**

After failed medical management (antihypertensives and antispasmodics) and onset of labor, an innovative “reverse-sequence cesarean myomectomy” (RCM) was performed with patient consent. This involved removing the giant lower uterine segment fibroid before delivering the fetus via C-section, utilizing a tourniquet.

**Outcomes:**

The RCM procedure was successful. The fibroid was removed, the baby delivered, and the C-section completed with only 400 mL blood loss. Both mother and infant had good outcomes, avoiding fetal removal difficulty and massive hemorrhage. Pathology confirmed leiomyoma. The approach utilized post-myomectomy uterine contraction for hemostasis and prevented the need for secondary surgery.

**Lessons:**

This case demonstrates that RCM is a safe, feasible, and innovative strategy for extreme cases where huge, strategically located fibroids (e.g., lower uterus) completely obstruct the birth canal. Its core advantages are: (1) solving the “unable to remove fetus” dilemma; (2) reducing bleeding risk via reverse timing (tumor first) and tourniquet; (3) avoiding a second surgery. RCM provides a valuable new option for managing these complex, high-risk pregnancies.

## 1. Introduction

Uterine fibroids are one of the most common comorbidities during pregnancy, with a reported prevalence ranging from 0.1% to 3.9%^[1]^ in pregnant women, and can even reach up to 10%.^[2]^ Pregnancy complicated by uterine fibroids poses significant risks to both maternal health and the fetus. These risks included preterm birth, hinder normal fetal movement within the uterus, increase the incidence of abnormal fetal positions, impair fetal development, cause premature rupture of membranes, disrupt regular uterine contractions, and elevate the risk of postpartum hemorrhage.^[3]^ Thus, uterine fibroids during pregnancy significantly impact maternal health, safety, and quality of life. Although ultrasound is the main diagnostic tool, magnetic resonance imaging can provide a more accurate assessment in complex cases. Surgical resection is the main treatment for symptomatic fibroids, but its role during cesarean section (C-section) is highly controversial due to concerns about incurable bleeding. Giant fibroids bring special challenges because of their space-occupying effect and abundant blood supply. This report aims to solve the gap in the huge lower part of the uterus or cervical fibroids that completely block the birth canal. In such cases, standard uterine fibroid removal after fetal delivery is anatomically not feasible. The implementation of the reverse sequence method we implemented in this report is determined by the size and location of the fibroid and the complete blockage of the birth canal. Considering the difficulty of surgery and the high risk of bleeding during the operation, it is very important to carry out rigourous evaluation and monitoring before and during the operation.

## 2. Case report

**Figure 1.**
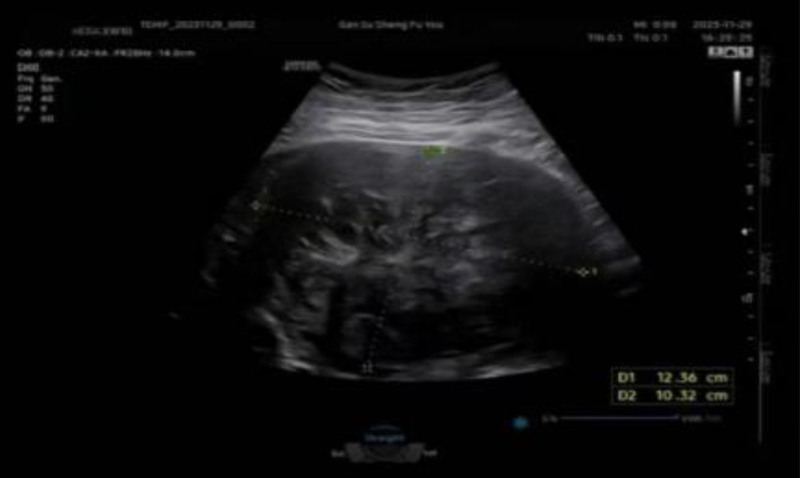
Obstetric ultrasound image at 36 weeks of pregnancy.

A 35-year-old woman (gravida 3 para 1) from Qinghai province was admitted to the Department of Obstetrics at Gansu Provincial Maternity and Child Health Care Hospital on January 30, 2024, due to “amenorrhea at 35 + 1 weeks of gestation and poorly controlled hypertension for 9 weeks.” Her past medical history included a 4-year history of type 2 diabetes and a 1-year history of hypertension. Last menstrual period was May 26, 2023. She reported irregular menses with no history of dysmenorrhea. She received regular antenatal care at an external hospital with suboptimal glycemic control. Initial hospital antenatal checkup at our hospital on January 30, 2024. Admission examination: blood pressure 163/103 mm Hg. Physical examination revealed no significant abnormalities in the heart or lungs. Gynecological examination: uterine fundus located 2 fingerbreadths below the xiphoid process. Fetal presentation was high and floating; fetal position unclear. A protruding mass approximately 5 cm in diameter was palpable 4 fingerbreadths above the pubic symphysis. Sterile vaginal examination: cervical length 2.5 cm, firm, central, closed; fetal presentation not palpable. Obstetric ultrasound: fetal size appropriate for gestational age. A hypoechoic mass measuring 149 × 141 × 93 mm was detected in the anterior uterine wall near the lower segment, extending superiorly to the mid-uterine body and inferiorly to the cervix. The mass had clear borders, heterogeneous internal echogenicity, and scattered punctate blood flow signals (see Fig. 1). She was admitted with diagnoses of “pregnancy complicated by uterine mass; chronic hypertension complicated by preeclampsia; diabetes complicating pregnancy; pregnancy with obesity; breech presentation; advanced maternal age.”

**Figure 2.**
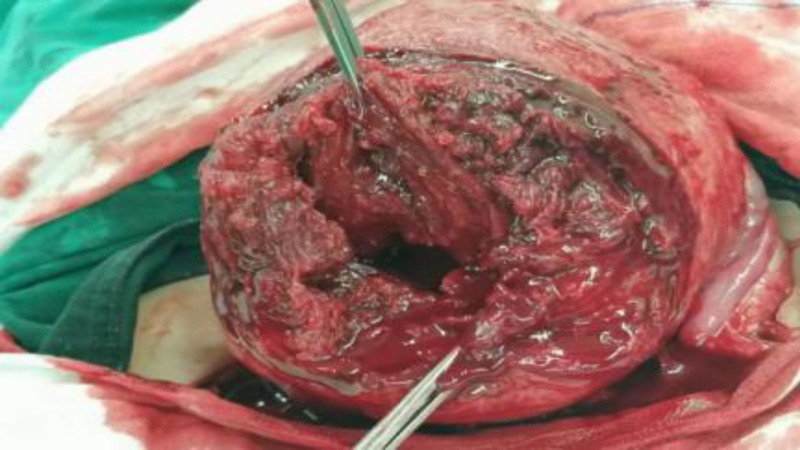
Myoma enucleation diagram.

**Figure 3.**
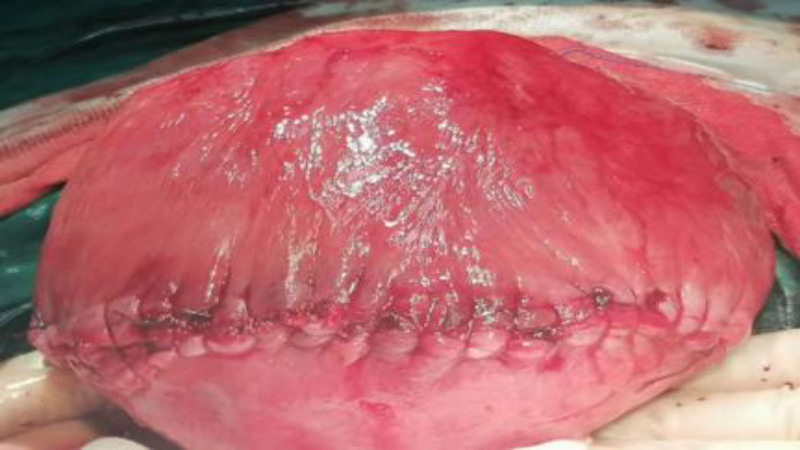
Uterus after suturing.

**Figure 4.**
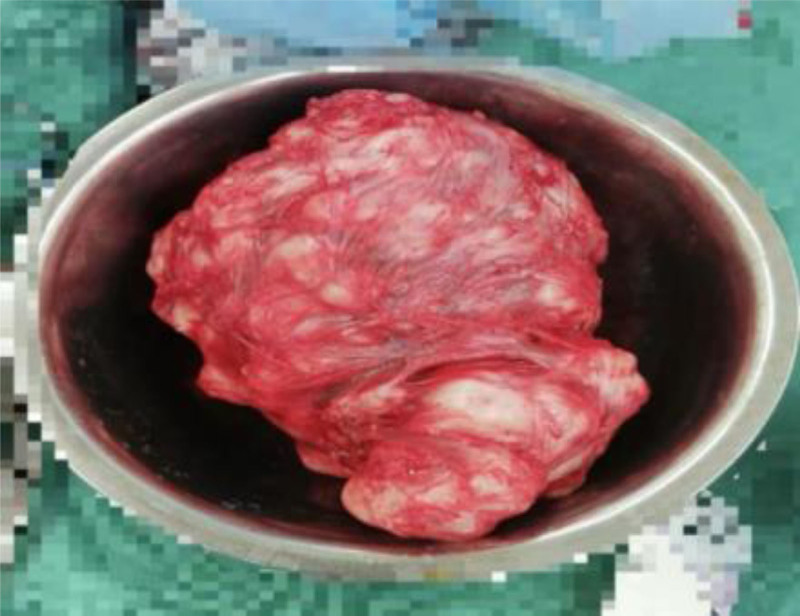
Enucleated myoma.

After admission, relevant laboratory tests were promptly completed, and management focused on controlling blood pressure and blood glucose. At 36 + 4 weeks of gestation, she developed regular lower abdominal pain. Ultrasound indicated amniotic fluid at the lower limit of normal. Due to significant blood pressure fluctuations and regular uterine contractions, prompt delivery was deemed necessary. Because the huge fibroids in the lower part of the uterus completely cover the inner opening of the cervix to block the descent of the fetus, vaginal delivery is impossible, and the delivery of the fetus by standard C-section is extremely dangerous. After multidisciplinary discussion, consensus is reached that it is inclined to perform reverse-sequence cesarean myomectomy (RCM) in the emergency department and remove the fibroid first before delivering the fetus. A transverse lower abdominal incision (3 fingerbreadths above the pubis) was made. Upon entering the abdominal cavity, a protruding mass approximately 5 cm in diameter was visible on the anterior lower uterine segment. Its surface was smooth and reddish, with no varicosities. Palpation could not delineate the fibroid boundaries or fetal parts. A transverse incision was made through the capsule over the protruding part of the mass, and the mass was bluntly dissected along the capsular edge. During dissection, the broad base of the mass made the procedure difficult. The dissection area was consequently enlarged, and the mass was completely enucleated (see Fig. 2). After removal, only the mucosal layer remained between the base of the tumor cavity and the uterine cavity, restoring the anatomical structure of the birth canal (see Fig. 3). The excised fibroid measured 17 × 17 × 7 cm and weighed 1100 g (see Fig. 4). Palpation superiorly along the tumor cavity identified the amniotic sac and fetal feet. The mucosal layer was incised, amniotomy performed, and a male infant was delivered by breech extraction by traction on both feet. Apply a temporary tourniquet to the lower part of the uterus to suture the uterine wound in layers, and the tumor cavity and the C-section incision are continuously sutured as a whole to prevent uterine deformation. The estimated blood loss during the operation is about 400 mL, without blood transfusion.The patient was discharged on postoperative day 4 in improved condition. Postoperative pathology confirmed a uterine leiomyoma.

## 3. Discussion

The definition of a giant uterine fibroid remains somewhat controversial but typically refers to a fibroid with a diameter ≥ 9 cm or weight ≥ 800 g, accounting for approximately 4% to 25% of fibroids complicating pregnancy.^[4]^ Due to their large size, giant fibroids are relatively uncommon clinically and pose significant management challenges. They can compress surrounding organs and tissues, leading to various clinical symptoms. During pregnancy, giant uterine fibroids may cause uterine atony, increasing the risk of postpartum hemorrhage. Therefore, pregnancy complicated by a giant uterine fibroid constitutes a serious threat to maternal and fetal health. Some studies suggest that uterine fibroids during pregnancy are a major factor contributing to poor pregnancy outcomes.^[5]^ This case introduces RCM to treat the huge lower uterine fibroids that completely block the birth canal. Traditional removal of uterine fibroids after fetal delivery is not feasible here. The reasons are: (1) the fibroid hinders the safe delivery of the fetus; (2) the fibroid is huge in size (>9 cm). If it is not removed, the uterus cannot be sutured safely, and there is a risk of bleeding due to weak uterine contractions; (3) standard uterine incision. The transverse fibroids will lead to uncontrollable bleeding. And RCM has significant advantages: (1) first, remove the obstructive mass and restore the anatomy of the birth canal. Using physiological hemostasis: the subsequent uterine contraction compresses the blood vessels at the base of the tumor cavity. It avoids tension suturing around large fibroids that have not been removed.

Should fibroids complicating pregnancy be removed during C-section? The current guidelines are cautious about removing the huge myoma in the lower segment fibroids during C-section^[6]^ primarily due to bleeding risks. However, recent meta-analysis shows that it is relatively safe in selective cases. Mollica et al^[6]^ conducted a prospective study of 106 pregnant women with fibroids. The study indicated that women who underwent myomectomy (n = 18), regardless of gestational age, had lower risks compared to conservatively managed women for miscarriage (0% vs 13.6%), premature rupture of membranes (5.6% vs 22.7%), preterm birth (5.6% vs 21.6%), and post-cesarean hysterectomy (0% vs 4.5%). However, uterine blood flow is significantly increased during pregnancy, and myomectomy at this time carries a risk of uncontrollable hemorrhage. Therefore, except for subserosal fibroids, fibroids near the uterine incision site, or cases of fibroid degeneration, concurrent myomectomy during C-section is generally not recommended. A meta-analysis by Pergialiotis et al,^[7]^ involving 3900 women (2301 undergoing concurrent cesarean myomectomy vs 1599 controls with cesarean only), found that the myomectomy group had a mild decrease in hemoglobin (mean difference 0.25 mg/dL, 95% CI: 0.06–0.45) and longer operative time (mean difference 13.87 minutes, 95% CI: 4.87–22.95). However, there were no statistically significant differences in transfusion rates (OR = 1.41, 95% CI: 0.96–2.07) or postoperative fever rates (OR = 1.12, 95% CI: 0.08–1.56) between the groups. Considering all factors comprehensively, the decision for myomectomy in this patient was based on: (1) the fibroid’s location in the lower uterine segment was large and unavoidable for fetal extraction; the uterine wall is thicker there, and an incision on the uterine body or fundus would be prone to bleeding. (2) Even after fetal delivery, the presence of a fibroid > 9 cm would make uterine closure extremely difficult without removing the fibroid. Forcing closure without myomectomy could hinder uterine contraction due to the large fibroid mass, potentially leading to postpartum hemorrhage. (3) The rapid growth of the fibroid during pregnancy raised concerns about degeneration or potential malignancy; removal during C-section could reduce risks of postoperative recurrence or malignant transformation. Overall, our RCM method is consistent with the principle of selective CM, but the challenge of completely blocking of the birth canal canal is solved by inverting the surgical order.

Studies^[8]^ suggest that for large fibroids (diameter > 5 cm) requiring removal during C-section, a vertical abdominal incision is preferable, and myomectomy should be performed after fetal delivery. Surgery should aim to minimize uterine trauma and bleeding based on the specific situation. A study of 200 pregnancies with fibroids^[9]^ found that trans-endometrial myomectomy is safe, with advantages of less bleeding, shorter operative time, and potential as a routine method for concurrent myomectomy during C-section. Another study^[10]^ noted that fibroids near the lower uterine segment incision can be removed directly from the incision edge without a separate incision; fibroids on the uterine body can be accessed via longitudinal, oblique, or transverse incisions directly over the fibroid core; multiple anterior wall fibroids may necessitate an “S”-shaped incision. Except for submucosal fibroids removed transcervically, the cesarean incision should be closed first before removing other fibroids.

In the above literatures (see Table 1), fibroid removal after fetal delivery typically causes greater uterine trauma. However, this case demonstrates fibroid removal before fetal delivery: the first reported use of reverse sequence surgery for lower uterine obstructive fibroids. This approach solves the core clinical dilemma of “no fetal extraction possible without fibroid removal.” The leiomyoma of the patient was located in the lower uterine segment and extended to the cervix, with a maximum diameter of 17 cm. The birth canal was completely blocked and the fetal presentation could not be connected. The leiomyoma in this position is rich in blood supply and adjacent to the bladder and ureter. The traditional C-section incision can not avoid the leiomyoma. If the fetus is forcibly removed through fibroids, it is likely to cause uncontrollable laceration bleeding. In this case, the reverse operation sequence was used, the myoma was completely removed, the lower segment of the anatomical structure was restored, and then the fetal membrane was broken. This strategy successfully avoided the risk of fetal removal due to myoma obstruction. The intraoperative blood loss was only 400 mL, which was significantly lower than the literature average of myoma in similar locations. Secondly, in view of the high-risk state that only the mucosal layer is left at the base of the tumor cavity and is connected with the uterine cavity, the lower uterine segment was ligated with a temporary tourniquet in this case to block the blood flow of the uterine artery and reduce bleeding from dissection; myomectomy cavity and C-section incision were sutured continuously as a whole to avoid uterine deformation caused by traditional step-by-step operation (Fig. 3). This combined technology verifies the effectiveness of the combined use of tourniquet and continuous suture technology, and provides new ideas for haemostasis of giant fibroids combined with thin-walled uterus.

**Table 1:** Literature comparison.

Study (yr)	Fibroid characteristics	Surgical approach	Blood loss (mL)	Limitations	Advantage of current case
Pergialiotis (2017)	Multiple, Avg. diam. 6.5 cm	Fetal delivery first, then myomectomy	520 ± 210	Primarily uterine body fibroids	Solution for lower segment fibroids
Dai Yan (2022)	Submucosal (transcervical route)	Fetal delivery first, then hysteroscopic removal	300 ± 50	Only suitable for submucosal fibroids	Solution for giant intramural fibroid
Sparić (2017)	Anterior wall (Avg. 8 cm)	Conventional CS incision removal	550 ± 180	Did not address lower segment fibroids	Reverse sequence + Tourniquet technique
This study	Lower segment, 17 cm	Myomectomy first, then fetal delivery	400	Extremely complex case	Solution for unavoidable obstructing fibroid

This report has inherent limitations. As a single case study, the method of RCM is limited in generality. The long-term outcome of uterine integrity or fibroid recurrence in future pregnancy is not clear, and its success depends greatly on a professional multidisciplinary team and meticulous preoperative planning. The outcome may be different in institutions with fewer resources. The lack of controlled cases makes it impossible to clearly attribute the low amount of bleeding entirely to the order of surgery or the specific hemostatic technique. Further prospective studies or case series are needed to verify the safety of RCM and improve the patient selection criteria.

This case confirmed that it can be safely implemented through strict indication control (myoma completely blocking the birth canal, unable to avoid the incision) and multidisciplinary plan (preoperative image positioning + blood preparation); the reverse-sequential operation makes the uterine contraction naturally compress the blood vessels in the tumor cavity, which has the advantage of hemostasis. Therefore, for the obstructive giant fibroids of the lower part of the uterus, the RCM in this case restores the anatomy of the lower part of the uterus after confirming the removal of the fibroids, so that the fetus can be delivered. It is possible that despite the complexity of the situation, no surgical complications have occurred, which provides a solution to the clinical dilemma of completely blocking the birth canal and not being able to remove the fetus. Although it is not a routine recommendation, RCM represents a key innovative option under a strict multidisciplinary program to address the specific challenge that giant fibroids completely hinder the delivery of the fetus during C-section. This case provides a basic example and technical framework for managing this rare but high-risk obstetric condition.

## Acknowledgments

The authors are grateful for the support from the Science.

## Author contributions

**Supervision:** Chengzhao Shu.

**Writing – original draft:** Jianfen Wang.

**Writing – review & editing:** Yan Dong.
